# Surfactant Maturation Is Not Delayed in Human Fetuses with Diaphragmatic Hernia

**DOI:** 10.1371/journal.pmed.0040237

**Published:** 2007-07-31

**Authors:** Olivier Boucherat, Alexandra Benachi, Bernadette Chailley-Heu, Marie-Laure Franco-Montoya, Caroline Elie, Jelena Martinovic, Jacques R Bourbon

**Affiliations:** 1 Institut national de la santé et de la recherche médicale (INSERM), Unité 841—Institut Mondor de Recherche Biomédicale, Créteil, France; 2 Université Paris 12, Faculté de Médecine, Créteil, France; 3 Université Paris-Descartes, Paris, France; 4 Maternité, Assistance Publique-Hôpitaux de Paris et Hôpital Necker-Enfants Malades, Paris, France; 5 Service de Biostatistique et Informatique Médicale, Assistance Publique-Hôpitaux de Paris et Hôpital Necker-Enfants Malades, Paris, France; 6 Service de Fœtopathologie, Assistance Publique-Hôpitaux de Paris et Hôpital Necker-Enfants Malades, Paris, France; National Heart and Lung Institute, United Kingdom

## Abstract

**Background:**

Pulmonary hypoplasia and persistent pulmonary hypertension account for significant mortality and morbidity in neonates with congenital diaphragmatic hernia (CDH). Global lung immaturity and studies in animal models suggest the presence of surfactant deficiency that may further complicate the pathophysiology of CDH. However, data about surfactant status in human fetuses with CDH at birth are contradictory. The lack of a chronological study of surfactant content in late pregnancy has been a significant limitation. The appropriateness of administering surfactant supplements to neonates with CDH is therefore a debated question.

**Methods and Findings:**

We investigated surfactant content in human fetuses with CDH compared to age-matched fetuses with nonpulmonary diseases used as controls. Concentrations of disaturated phosphatidylcholine and surfactant proteins were found to be similar at a given stage of pregnancy, with both components showing a similar pattern of increase with progressing pregnancy in fetuses with CDH and in control fetuses. Thyroid transcription factor 1, a critical regulator of surfactant protein transcription, similarly displayed no difference in abundance. Finally, we examined the expression of three glucocorticoid-regulated diffusible mediators involved in lung epithelial maturation, namely: keratinocyte growth factor (KGF), leptin, and neuregulin 1 beta 1 (NRG1-β1). KGF expression decreased slightly with time in control fetuses, but remained unchanged in fetuses with CDH. Leptin and NRG1-β1 similarly increased in late pregnancy in control and CDH lungs. These maturation factors were also determined in the sheep fetus with surgical diaphragmatic hernia, in which surfactant deficiency has been reported previously. In contrast to the findings in humans, surgical diaphragmatic hernia in the sheep fetus was associated with decreased KGF and neuregulin expression. Fetoscopic endoluminal tracheal occlusion performed in the sheep model to correct lung hypoplasia increased leptin expression, partially restored KGF expression, and fully restored neuregulin expression.

**Conclusions:**

Our results indicate that CDH does not impair surfactant storage in human fetuses. CDH lungs exhibited no trend toward a decrease in contents, or a delay in developmental changes for any of the studied surfactant components and surfactant maturation factors. Surfactant amounts are likely to be appropriate to lung size. These findings therefore do not support the use of surfactant therapy for infants with CDH. Moreover, they raise the question of the relevance of CDH animal models to explore lung biochemical maturity.

## Abstract 2

In an autopsy study of human fetuses, Jacques Bourbon and colleagues report that pulmonary surfactant content is not decreased in congenital diaphragmatic hernia.

## Editors' Summary

**Background.:**

Congenital diaphragmatic hernia (CDH), a fetal malformation in which the abdominal organs are displaced into the chest cavity, occurs in approximately one out of 3,000 live births and accounts for approximately 8% of major birth defects. The displaced lungs tend to be underdeveloped at birth, and decreased lung function is a major cause of sickness and death in affected babies.

Pulmonary surfactant, a substance naturally produced by cells in the lungs in the weeks before birth, is necessary for normal breathing. Surfactant acts to keep the walls of the lung's airspaces from collapsing onto each other, much as detergent can keep the walls of a moist plastic bag from sticking together.

**Why Was This Study Done?:**

The specific aspects of lung immaturity that cause decreased function in babies with CDH are not fully understood. In particular, it has been unclear whether abnormally low levels of pulmonary surfactant contribute to the lung problems caused by CDH. Some studies in sheep or rat models of CDH have found that surfactant levels are abnormally low, while others have found normal or even increased levels of surfactant. Studies in human CDH have also shown controversial results, and few have investigated surfactant production in lung tissue itself.

Doctors can artificially increase babies' pulmonary surfactant levels if necessary through the instillation of exogenous surfactant material, but doing so involves some risk. This is a routine treatment with demonstrated benefit in premature neonates with normal, although not fully developed, lungs. Pulmonary surfactant is sometimes given also to babies with CDH, but in this particular instance, the benefit of this practice is unclear. The authors of this study wanted to determine whether or not babies with CDH really have low levels of pulmonary surfactant.

**What Did the Researchers Do and Find?:**

The researchers performed autopsies to study lung tissue from human fetuses in pregnancies that ended in fetal death, were terminated for medical reasons, or resulted in death immediately after birth. They compared surfactant production in 16 fetuses with CDH to that in 33 fetuses with conditions not involving the lungs. They found that, taking fetal age into account, surfactant was present at similar levels in lung tissue from both groups, and that production of surfactant components and other factors involved in lung maturation was not delayed in CDH.

The researchers also studied sheep fetuses with surgically induced CDH, a model that has been used in the past to study lung development in CDH. In contrast with the findings in humans, they found that factors stimulating surfactant production were decreased in sheep with surgically induced CDH.

**What Do These Findings Mean?:**

These findings suggest that surfactant deficiency is not a major contributor to lung problems in babies with CDH, and that further clinical research may be appropriate to determine whether administering surfactant to these babies is beneficial. The findings also suggest that commonly used animal models of CDH may be of limited relevance to human CDH.

**Additional Information.:**

Please access these Web sites via the online version of this summary at http://dx.doi.org/10.1371/journal.pmed.0040237.

- Read the related Perspective on this article by Marcus Davey
- Information from the MedlinePlus Encyclopedia (US National Library of Medicine) on diaphragmatic hernia
- Article from eMedicine on congenital diaphragmatic hernia
- Wikipedia entry on pulmonary surfactant (note: Wikipedia is an online encyclopedia that anyone can edit)

## Introduction

Congenital diaphragmatic hernia (CDH) is a developmental abnormality that affects one in 2,500–5,000 live births, depending on the studies, and accounts for 8% of all major congenital anomalies. CDH restricts fetal lung development. The resulting lung hypoplasia and hypertension have dramatic consequences at birth, and the disease continues to cause high rates of mortality and morbidity despite recent progress in neonatal care [1,2]. Data from human fetuses with CDH, as well as data from animal models, indicate global lung immaturity with respect to fetal age, including reduced bronchial branching, decreased acinar complexity, thickened septa, and reduced vascularization [3]. Although this concept is debated, lung immaturity in CDH has also been suggested to involve the surfactant system.

Pulmonary surfactant, which is a lipid–protein complex produced by alveolar type II (ATII) cells, prevents alveolar collapse during the breathing cycle by reducing surface tension at the air–liquid interface [4]. Surfactant deficiency, the principal cause of which is premature birth, results in neonatal respiratory distress. The major component of surfactant is a surface-active phospholipid, disaturated phosphatidylcholine (DSPC). Four characteristic surfactant proteins (SPs) include the collectins SP-A and SP-D, which play a role in innate immunity and regulate surfactant homeostasis [5], and the hydrophobic proteins SP-B and SP-C, which augment the surface properties of phospholipids [6]. During fetal development, the production of surfactant is not abundant until late pregnancy. The developmental regulation of surfactant synthesis includes both hormonal control and mesenchymal–epithelial interactions [7]. Under glucocorticoid stimulation, fetal lung fibroblasts release factors that promote ATII cell maturation [8]. Three diffusible mediators, including fibroblast growth factor 7 (also known as keratinocyte growth factor [KGF]) [9,10], leptin [11], and neuregulin 1 beta 1 (NRG1-β1) [12,13] have recently been proposed to mediate this stimulating activity.

The prevailing opinion that the CDH lung is surfactant deficient is principally based on the immature morphological aspect of the parenchyma and on reports of diminished surfactant content in animal models of CDH. The surfactant system has been repeatedly reported to be deficient in two classical models of CDH, the surgically created diaphragmatic hernia (sDH) in the fetal sheep [14–17] and the nitrofen model in the rat fetus [18–20]. This conclusion was based on a variety of determinations, including tissue and lavage-fluid phospholipids, lavage-fluid SPs, tissue SP and SP-transcript levels, and dynamic surface-tension measurement. However, conflicting results have been published indicating no change in alveolar surfactant composition [21], unchanged or even increased SP expression with a higher proportion of cells expressing SP in the nitrofen model [22,23], and increased SP expression in the ovine model [24].

Controversy persists regarding whether or not human infants with CDH are surfactant deficient. Whereas some studies concluded that surfactant components failed to increase in amniotic fluid [25], or that alveolar SP-A and DSPC were decreased 75% and 50%, respectively, despite a normal rate of DSPC synthesis [26,27], others reported no significant change in amniotic fluid [28] or lung lavage fluid [29] phospholipids. Immunohistochemical detection of SP-A indicated weak labeling and a reduced proportion of positive cells, suggesting the possibility of delayed ATII cell maturation [30]. The controversy over surfactant deficiency is likely to have arisen from a variety of causes, including the use of various methodological approaches arriving at different conclusions, and the use of different therapeutic practices. First, a variety of biological materials have been explored, including amniotic fluid [25,28], tracheal aspirates [26], and lavage fluid [29], and different maturity tests with debatable values have been used, including evaluation of DSPC [25,26], lecithin-to-sphingomyelin ratio, and levels of phosphatidylglycerol [26,27] and SPs [26,30]. Second, it is possible that changes were induced by prenatal exposure to glucocorticoids [31] or by neonatal care methods (e.g., ventilation and extracorporeal membrane oxygenation) [26]. Third, there is a paucity of studies of CDH human lung tissue that might have helped to resolve the question.

Clarifying this question is important insofar as decisions have to be made as to whether or not infants with CDH should be supplemented with surfactant at birth [32–34]; this issue is particularly important because the use of exogenous surfactant in CDH is routine in several neonatal centers. No actual quantitative determination of surfactant in human CDH lung tissue is available. Taking advantage of access to fetal human CDH lung tissue samples and lung tissue samples from fetuses with nonpulmonary diseases used as controls, we reappraised the question through the determination of the pulmonary content of major surfactant components, including DSPC and SPs, and their accumulation with progressing pregnancy in human fetuses with CDH. In parallel, we examined the expression of mesenchymal factors involved in pulmonary epithelial differentiation and maturation. We also evaluated these factors in the sheep model with sDH, in the presence or absence of tracheal occlusion (TO). Also designated as the PLUG method, TO is a novel approach to treating CDH lung hypoplasia currently under trial in humans [35].

## Methods

### Human Lung Tissue

Postmortem lung tissue samples were obtained at autopsy after medical termination of pregnancy or neonatal death, with signed, informed consent from the parents. Terminations were performed according to the July 1994 French legislation, and the study was undertaken with the agreement of the institutional Ethical Committee. The prenatal diagnosis of CDH was made by echography, and was confirmed by postmortem examination. Reduced lung weight and consistent histological appearance confirmed the diagnosis of pulmonary hypoplasia (lung to body weight ratio < 0.015 before 28 wk of pregnancy, and < 0.012 thereafter [Table 1]). Fetuses were checked for other malformations and for the presence or absence of associated chromosomal abnormality. The lung ipsilateral to the hernia was used. Lungs from fetuses with nonpulmonary diseases were used as controls; they appeared to be histologically normal by postmortem examination and were not hypoplastic. Detailed clinical data are depicted in Table 2. Preservation of RNAs in human lung tissue samples was insufficient and inconsistent, and therefore molecular study at the pre-translational level could not be performed. Fetal age (postconception) is used throughout the paper.

**Table 1:**
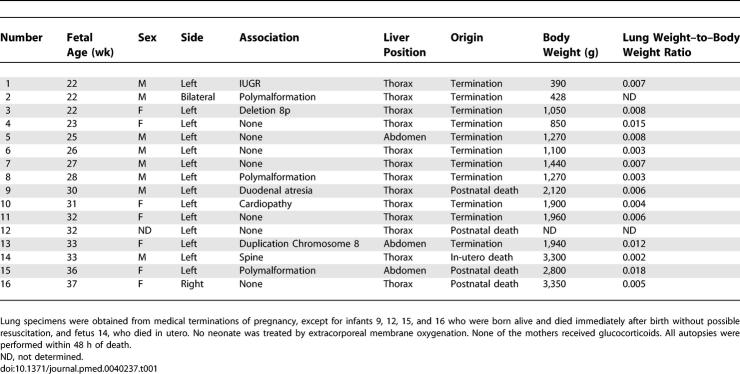
Characteristics of CDH Fetuses

**Table 2:**
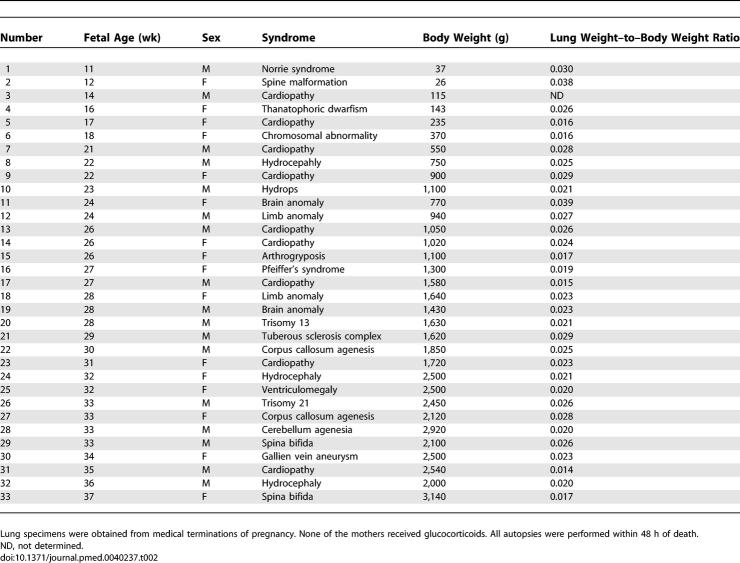
Characteristics of Fetuses with Nonpulmonary Diseases Used as Controls

### Surgical Model of CDH in the Sheep Fetus and TO

All animal experiments were performed with the authorization of the French Ministry of Agriculture. Surgical procedures have been extensively described elsewhere [36]. Biological samples were from the same animals as those in previous studies [16,17,37]. In brief, three groups of Pre-Alp sheep fetuses (full term = 145 d) were studied. The first group, designated the surgical diaphragmatic hernia (sDH) group (*n* = 5), was obtained by creating a left-sided diaphragmatic hernia in utero at 85 d of gestation. The second group, designated the sDH + TO group (*n* = 3) was obtained by creating sDH at 85 d as in the first group, then TO at 120 d with a latex balloon inserted via noninvasive endoscopic technique. The third group, designated the control group (*n* = 6), consisted of twin fetuses that were sham-operated for sDH. Fetuses were retrieved by cesarean section at 139 d of gestation, and lung tissue was immediately frozen and stored at −80 °C.

### DSPC Determination

The technique has been described in detail previously [16]. Briefly, lipids were extracted from lung homogenates by chloroform:methanol:water, 1:2:0.8 (vol/vol/vol). After addition of a trace amount of labeled dipalmitoylphosphatidylcholine (GE Healthcare Life Sciences, https://www1.gelifesciences.com) for recovery determination, total phosphatidylcholine (PC, whole-lipid extract) and DSPC (osmium tetroxide–treated lipid extract) were separated by thin-layer chromatography. PC and DSPC were eluted from the gel and divided into aliquot fractions for scintillation counting and for phosphate determination after sample mineralization.

### Lung DNA

DNA was determined on residual pellet from delipidated homogenates by the colorimetric diphenylamine method [38]. DPSC was normalized for DNA concentration.

### Western Blot Analysis

Human lung tissues were homogenized in RIPA buffer containing protease inhibitors (Roche Diagnostics, http://www.roche.com). Proteins (60 μg) were electrophoresed on a 12% SDS-polyacrylamide gel and transferred onto a polyvinylidene fluoride membrane (Millipore, http://www.millipore.com), then stained with Ponceau S dye (Sigma, http://www.sigmaaldrich.com). After blocking with 5% nonfat dry milk in Tris-buffered saline containing 0.05% Tween-20 (TTBS) at room temperature for 2 h, membranes were exposed for 2 h to one of the following antibodies: anti-SP-A (gift from M. Post, Hospital for Sick Children, Toronto, Ontario, Canada), anti-SP-B (gift from J. A. Whitsett, Cincinnati Children's Hospital Medical Center, Cincinnati, Ohio, United States), anti-proSP-C (Chemicon International, http://www.chemicon.com), anti-SP-D (BMA Biomedicals, http://www.bma.ch), anti-thyroid transcription factor 1 (TTF1, Santa Cruz Biotechnology, http://www.scbt.com), and anti-NRG1-β1 (R&D Systems, http://www.rndsystems.com), all diluted in 2% nonfat dry milk in TTBS, then incubated for 1 h with the appropriate secondary IgG peroxidase-conjugated antibody. After washing in TTBS, membranes were incubated for 1 min in chemiluminescent detection reagent (ECL, GE Healthcare Life Sciences), before exposure to Kodak BioMax MS film (GE Healthcare Life Sciences) for 2 min. Densitometry analysis of blots was performed using NIH image software.

### ELISA

KGF and leptin concentrations in human fetal lung tissue were assessed with commercially sensitive and specific ELISA (R&D Systems), following the manufacturer's instructions, and normalized to total proteins. The intra-assay coefficient of variance was less than 5%.

### RNA Isolation and Reverse Transcription

Total RNA was extracted from fetal sheep lung tissue using Trizol reagent (Invitrogen, http://www.invitrogen.com) according to the manufacturer's instructions. The pelleted RNA was dissolved in sterile water and quantified by absorbance at 260 nm (BioPhotometer, Eppendorf, http://www.eppendorf.de). RNA quality and integrity were confirmed after electrophoresis of 1 μg of each sample in 1% agarose gel. RNAs were reverse-transcribed into cDNAs using 2 μg of total RNA, Superscript II reverse transcriptase, and random hexamer primers (Invitrogen), according to the manufacturer's protocol.

### Determination of Partial Ovine Neuregulin (NRG) cDNA Sequence

cDNAs were reverse-transcribed from sheep lung total RNAs with Superscript II reverse transcriptase and random hexamer primers (Invitrogen) as above. Amplification of the partial cDNA sequence for NRG was performed using sense primer 5′-TCAGAACTTCGCATTAGCAAAGC-3′ (bovine NRG–specific sequence) and antisense primer 5′-GGGAGTGGACGTACTGTAGAAGCT-3′ (bovine/human NRG–specific sequence). Amplification was performed through 35 cycles (1 min at 94 °C, 1 min at 59 °C, and 1 min at 72 °C). The amplified sequence was purified with QIAquick PCR purification kit (Qiagen, http://www.qiagen.com) before being sequenced with an ABI 3130 XL gene analyzer (Applied Biosystems, http://www.appliedbiosystems.com). The expression level of the transcripts was studied by real-time PCR in ovine samples using this sequence; because the transcripts correspond to common sequences for various NRGs, they are hereafter designated NRG mRNAs without isoform precision.

### Real-Time PCR

A 20-μl mix, containing 12.5 μl of Powerful SYBR Green PCR Master Mix (Applied Biosystems), 900 nM forward primer, and 900 nM reverse primer, was prepared for performing real-time PCR. Primers were designed to be intron-spanning to avoid co-amplification of genomic DNA, using Primer Express software (Applied Biosystems). Primer sequences are reported in Table 3. Reverse-transcribed cDNA (25 ng) was added to the PCR mix to a final volume of 25 μl. Real-time PCR was performed on an ABI Prism 7000 device (Applied Biosystems) using the following protocol: initial denaturation (10 min at 95 °C), then a two-step amplification program (15 s at 95 °C followed by 1 min at 60 °C) repeated 40 times. Melt-curve analysis was used to check that a single specific amplified product was generated. Real-time quantification was monitored by measuring the increase in fluorescence caused by the binding of SYBR Green dye to double-stranded DNA at the end of each amplification cycle. Relative expression was determined using the ΔΔCt (threshold cycle) method of normalized samples (ΔCt), in relation to the expression of a calibrator sample, according to the manufacturer's protocol. Each PCR run included a no-template control and a sample without reverse transcriptase. All measurements were performed in triplicate.

**Table 3:**
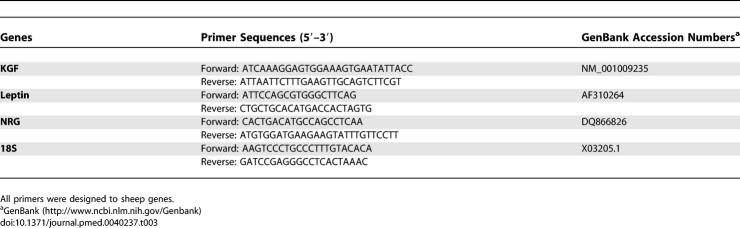
Primer Sequences for Real-Time PCR

### Statistical Analysis

Relationships between biological variables and fetal age were analyzed using linear-regression models. When appropriate, a quadratic term for time was introduced to fit an exponential increase with fetal age. Main effects and interaction terms were tested in order to compare biological values in human fetuses with CDH versus fetuses with nonpulmonary diseases used as controls (control fetuses). Sensitivity analyses were performed when outliers were present.

For NRG1-β1, SP-A, SP-B, proSP-C, and SP-D, comparison between human fetuses with CDH and control fetuses was made using the paired Mann-Whitney U-test. For these variables, except for NRG1-β1 in control lungs, regression models were not performed because of the restricted number of determinations.

For sheep data, multiple group comparisons were made using the Kruskal-Wallis test, and two-group comparisons were made using the Mann-Whitney U-test.

A *p*-value of 0.05 was considered to be the limit of statistical significance. All analyses were performed using R statistical software (http://cran.r-project.org).

## Results

### DSPC and SPs in Human Fetal Lungs

**Figure 1:**
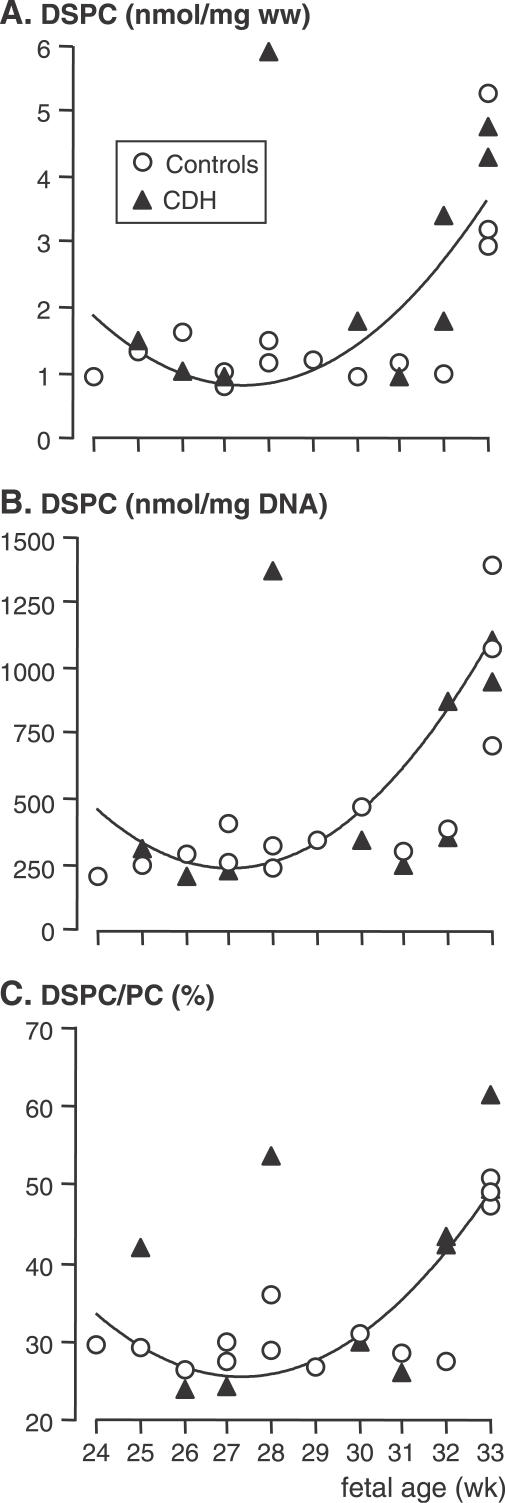
DSPC in Human Fetal Lungs DSPC was normalized to lung wet weight (ww) (A) and lung DNA (B), and expressed as a ratio of DSPC to total PC (i.e., DSPC plus unsaturated PC) (C). A rise in DSPC concentration and in the ratio of DSPC to total PC, which is indicative of an enhanced rate of surfactant accumulation, was observed at fetal age 32–33 wk in CDH as well as in control fetal lungs. There were no differences in the data between the groups, whatever the considered fetal age. DSPC displayed the same exponential increase in control lungs and CDH lungs with fetal age (*p*-value for quadratic term < 0.01).

DSPC concentration was determined in lung tissue samples from ten fetuses with CDH, ranging from 24 to 33 wk of pregnancy, and in 14 age-matched control fetuses, and normalized for lung DNA concentration. Over the studied period, DSPC concentration displayed a significant exponential increase with fetal age in control lungs and in CDH lungs (*p*-value for quadratic term < 0.01). The evolution of lung DSPC concentration did not differ significantly between fetuses with CDH and control fetuses when normalized either to lung wet weight (*p*-values for groups and interactions > 0.20; Figure 1A) or to lung DNA (*p*-values for groups and interactions > 0.25; Figure 1B), with approximately a 5-fold increase observed at 33 wk in both groups. Similarly, the ratio of DSPC to total PC increased in both groups at 33 wk (*p* < 0.01), which was indicative of an accelerating rate of maturation, and did not differ among groups (*p*-values for groups and interactions > 0.1; Figure 1C). Because of abnormally high ratios (outliers), one CDH fetus was excluded from the three analyses and another fetus was excluded from the DSPC/total PC analysis.

**Figure 2:**
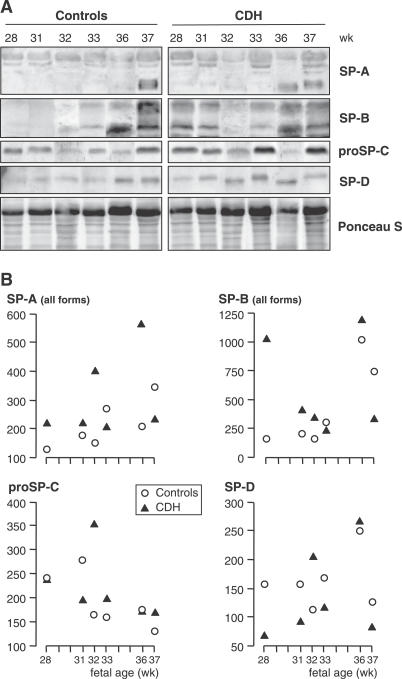
SPs in Human Fetal Lungs (A) Western blots: Samples were electrophoresed by SDS-PAGE, transferred, and successively incubated with specific anti-SP antibodies. (B) Densitometric analysis normalized by Ponceau S for gel loading (arbitrary units). SPs were detected at all stages. SP-A monomers were extremely faint before 36 wk, when they increased sharply (A) resulting in an increase in total amount (B). SP-B also increased markedly at 36–37 wk, ProSP-C did not display developmental changes, and SP-D showed a weak increase (A, B). There were no obvious differences between CDH and control lungs for any SP, with developmental changes occurring at the same fetal ages, the only exception being presentation of a high SP-B level in one CDH fetus, aged 28 wk.

Expression of SP-A, SP-B, SP-D, and proSP-C was evaluated at the post-translational level by Western blot analysis. Six pairs of CDH and age-matched control lungs ranging from 28 to 37 wk of pregnancy (i.e., all in saccular-alveolar stages) were studied comparatively. No obvious difference was observed for any SP between CDH and control lungs at any stage (Figure 2A). Densitometry indicated CDH values within the range of control values (*p*-values for the four comparisons > 0.40; Figure 2B). Low molecular weight monomeric forms of SP-A were detected in significant amounts only from 36–37 wk in each group, and were hardly in evidence at younger stages. Nonreducible di- and trimeric forms were detected at all stages, with no change with progressing pregnancy. SP-B was detected early, but was markedly increased also at 36–37 wk in both groups. The only difference between the groups was a higher SP-B content in the 28-wk fetus with CDH; it should be emphasized that this fetus also displayed a high DSPC concentration in Figure 1. ProSP-C showed no marked developmental changes, and SP-D tended to increase slightly in both groups.

### TTF1 in Human Fetal Lungs

**Figure 3:**
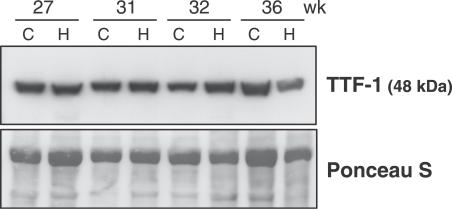
TTF1 Protein in Human Fetal Lungs Western blot was performed in lung samples of fetuses with CDH (lanes labeled H) and in fetuses with nonpulmonary diseases used as controls (lanes labeled C) at four fetal ages (Ponceau S stain was used as loading control). No differences were observed between CDH fetuses and age-matched control fetuses.

In parallel with SP expression, we evaluated by Western blot the expression of TTF1, a transcription factor known to play a major role in various aspects of fetal lung development, including as a positive regulator of surfactant–protein gene-promoter activity [39]. Consistent with our findings on SP expression, at no stage was any obvious difference found between fetuses with CDH and control fetuses (Figure 3).

### KGF, Leptin, and NRG1-β1 in Human Fetal Lungs

**Figure 4:**
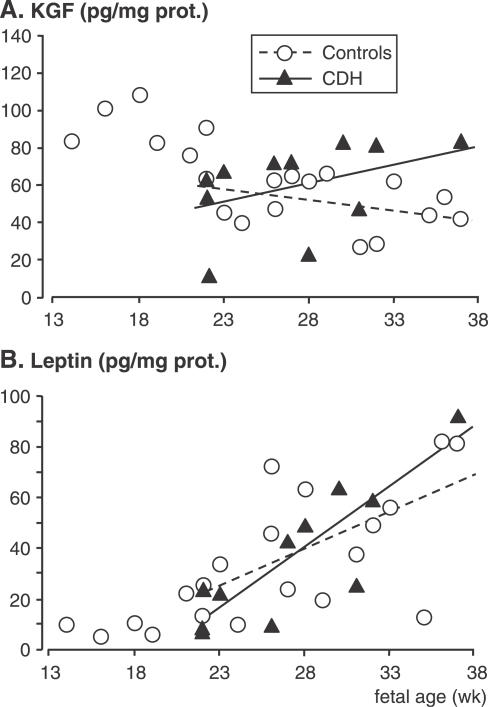
KGF and Leptin in Human Fetal Lungs ELISAs were performed on the lungs of 20 fetuses with nonpulmonary diseases used as controls, ranging from 14 to 37 wk of pregnancy, and on 11 fetuses with CDH ranging from 22 to 37 wk. Individual values are shown. Linear regression was performed for the period 22–37 wk when samples were available for both groups. KGF (A) displayed different profiles according to the groups concerned, with a negative slope for control lungs and a positive slope for CDH lungs (−1.17 versus 2.23, respectively, *p* = 0.03). Leptin assay (B) evidenced little change in control lungs before 22 wk; a similar linear increase occurred between 22 and 37 wk with no difference between groups (slope = 4.82 versus 2.60 for controls and CHD samples, respectively, *p* = 0.18).

**Figure 5:**
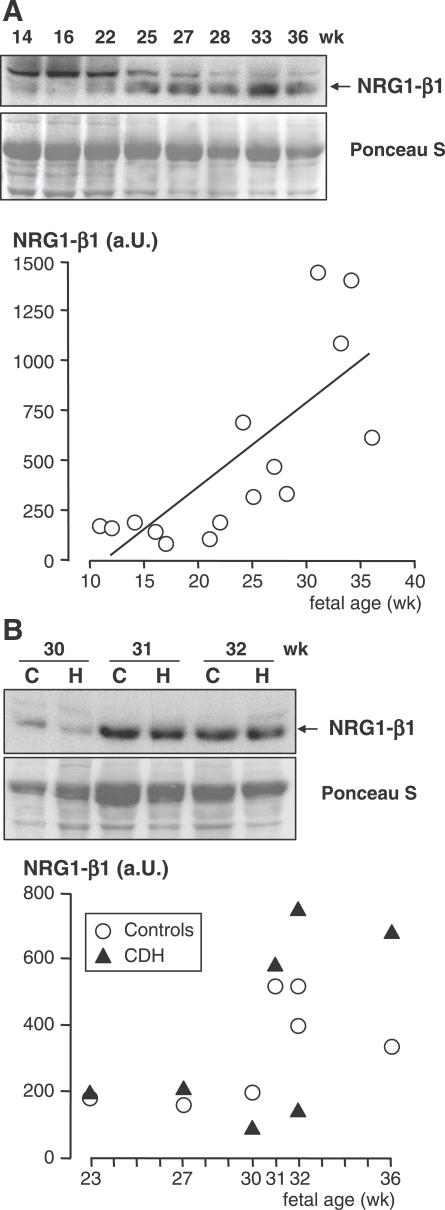
NRG1-β1 in Human Fetal Lungs (A) Developmental changes in control lungs were studied from 11 to 36 wk of pregnancy. Upper: representative Western blot. A 45-kDa band corresponds to NRG1-β1 (the 55-kDa band is nonspecific [61]). Lower: densitometric analysis (arbitrary units [a.U.]) in 15 individuals showing a significant linear increase (*r* = 0.75, *p* = 0.001). (B) Comparative study in seven pairs of age-matched CDH (lanes labeled H) and control (lanes labeled C) lungs. Upper: representative Western blot. Lower: densitometric analysis; there was no significant difference in expression level between CDH and control lungs (*p* = 0.58).

The expression of three growth factors involved in maturation control of ATII cells, namely KGF, leptin, and NRG1-β1 proteins, was studied in human lung samples at the post-translational level. In control lungs, KGF concentration decreased by about 50% between 14 and 38 wk (*r* = −0.71, *p* < 0.001). From 22 wk, when CDH samples were available, CDH and control lungs displayed statistically different KGF profiles, with a negative slope for control lungs and a positive slope for CDH samples (−1.17 versus 2.23, respectively, *p* = 0.03; Figure 4A). Conversely, the leptin profiles did not show any differences between the two groups (slope was 4.82 for controls versus 2.60 for CHD samples, *p* = 0.18; Figure 4B). Leptin concentration remained at around 10 pg/mg of protein until 20 wk, and increased linearly from 22 to 38 wk to reach approximately 80–90 pg/mg of protein at the later stages (Figure 4B). In control lungs, NRG1-β1 concentration remained unchanged until 22 wk, when it then increased sharply. At 33–35 wk, it reached 7-fold the level observed at early stages (Figure 5A). Despite the apparent exponential distribution of the values, it did not display a significant exponential increase (*p*-value for quadratic term = 0.25), whereas a significant linear correlation was found (*r* = 0.75, *p* = 0.001). No difference was found for NRG1-β1 between CDH lungs and age-matched control lungs, as evaluated in seven pairs aged 23 to 36 wk (*p* = 0.58; Figure 5B).

### KGF, Leptin, and NRG in the Ovine sDH Model, and Effects of TO

**Figure 6:**
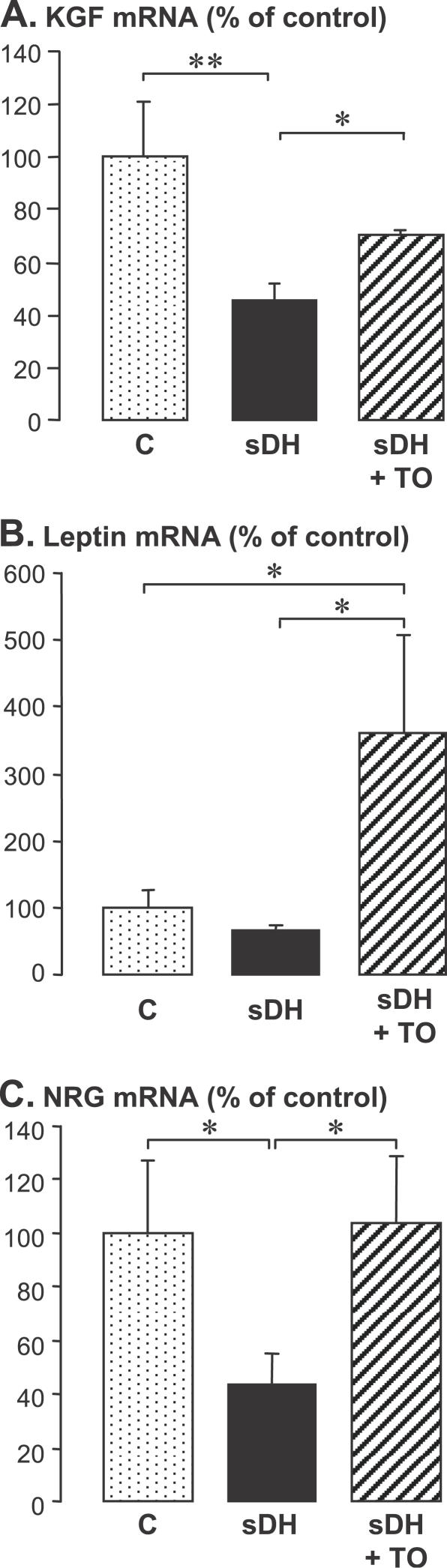
KGF, Leptin, and NRG mRNAs in Ovine Fetal Lungs RT followed by real-time PCR analysis; mean ± standard error of the mean on six, five, and three individual samples, respectively, in sham-operated control fetuses (C), in a surgical model of CDH (sDH), and in sDH with subsequent tracheal occlusion (sDH + TO). The Kruskal-Wallis test was used for multiple-group comparison, with two-group comparisons made by Mann-Whitney U-test (*p < 0.05 and **p < 0.01). (A) KGF mRNA level was reduced by half by sDH and was partially restored by sDH + TO. (B) Leptin mRNA was not affected by sDH, whereas TO enhanced expression to 3.5 times the control level. (C) NRG mRNA level was decreased by 60% by sDH and was restored by sDH + TO.

The three growth factors were analyzed in fetal sheep lungs at the pre-translational level by real-time PCR. KGF mRNA was significantly decreased in the sDH group, and its expression level was partially restored by TO (Figure 6A). The leptin mRNA level was not significantly affected by sDH, but TO enhanced expression to 3.5 times the control level (Figure 6B). Determination of the partial sequence of ovine NRG did not allow the NRG1-β1 mRNA isoform to be selectively determined by real time PCR because the partial sequence is common to various isoforms. The mRNA level determined using this sequence and designated NRG was diminished to 42% of control value in the sDH group, and was restored to control level in the sDH + TO group (Figure 6C).

## Discussion

A persistent question regarding the care of neonates with CDH is whether these infants are effectively surfactant deficient. The question of whether to administer surfactant to infants born with CDH is not trivial, as this therapy can transiently compromise gas exchange. Information about surfactant status in humans is limited, has been obtained by indirect approaches, and has remained controversial. We reappraised the question through the ontogenetic study of surfactant directly in CDH lung tissue samples. We showed that, contrary to current opinion, surfactant accumulation occurs in CDH lungs at the normal time and rate.

The postmortem collection of tissue samples was a limitation of this study and prevented us from studying expression at the pre-translational level. Investigations in humans also raise the question of control appropriateness, since only lung samples from subjects with nonpulmonary diseases could be used as controls. A previous demonstration of the differences between CDH and control lungs, considering other parameters, nevertheless validated the approach [40].

Developmental changes in the concentration of DSPC, the major surfactant component, were identical in CDH and control lung tissue samples, with a marked increase in late pregnancy. Surfactant-storing lamellar bodies appear in the human fetus at around 20 wk [41]. The rise observed for DSPC at around 33 wk therefore corresponds to an increased rate of accumulation, not to the initiation of surfactant synthesis. Although DSPC is present also in other lung cell membrane compartments, the marked rise in concentration and in the ratio of DSPC to total PC is illustrative of increasing surfactant content. It is therefore of particular significance that these parameters increased at the same fetal age in control and CDH lungs.

Also indicative of alveolar cell maturation is the increase in both SP-A and SP-B that was observed at the same time in both groups. Although the available amount of tissue from the 37-wk CDH lung was not sufficient for studying DSPC concentration, our results indicate similar timing for DSPC and SP accumulation. ProSP-C is of particular interest because, contrary to other SPs that are also expressed in bronchiolar Clara cells, it is a specific marker of ATII cells [42]. The comparable concentrations of proSP-C in CDH and non-CDH lungs therefore indicate similar ATII cell-maturation status in both instances. No developmental change was observed for proSP-C because it does not accumulate in the developing lung, unlike mature proteins [43]. Considering the role of TTF1 as a transcription regulator of SPs [39], the absence of any change in SP content is fully consistent with unchanged TTF1 protein abundance between CDH and control lungs, and with the comparable staining for TTF1 that has been observed previously [44].

Previous investigations about surfactant status in CDH have led to controversial data, which have left inconclusive the question of primary surfactant deficiency. Some studies that appear consistent with the present findings showed no change in surfactant content of amniotic fluid [28], or in bronchoalveolar lavage [29], from infants with CDH, whereas others reported decreased surfactant in amniotic fluid [25], or reduced levels of DSPC and SP-A in tracheal aspirates, from ventilated infants with CDH [26]. The present study, which, to our knowledge, is the first to appraise the question through the determination of surfactant in human lung tissue, indicates normal timing and a normal rate of surfactant accumulation in the CDH lung ipsilateral to the hernia.

There are several possible explanations for the discrepancies highlighted by previous investigations. First, although lung concentrations of surfactant components are normal, the total lung surfactant content is decreased in the CDH lung as a consequence of lung hypoplasia. This may account for the decreased surfactant levels measured in amniotic or lavage fluids. Nevertheless, the amount of surfactant is probably appropriate for lung size and alveolar surface area. Second, ventilation may have influenced surfactant composition and/or concentration. It has indeed been reported that ventilation alters alveolar surfactant in rat pups [45] and that ventilation strategies interfere differentially with surfactant metabolism in injured lungs [46]. Moreover, DSPC turnover was found to be more rapid in infants with CDH, presumably reflecting an increase in catabolism or recycling of DSPC [27]. Together, these factors may lead to a secondary surfactant deficiency acquired after birth, which may in turn account for a reduced tracheal aspirate content in CDH despite a normal amniotic fluid content.

In contrast with our findings in the human lung, surfactant deficiency has been repeatedly reported in the sheep [14–17] and rat [18–20] models of CDH. To further explore underlying mechanisms that may account for this discrepancy, we investigated comparatively in human and ovine lungs the expression of paracrine factors known to control ATII cell maturation. We used samples from the same sheep lungs as in previous studies [16–17]. A wide array of growth factors has been reported to stimulate surfactant production. We studied three potent stimulating factors—KGF, leptin, and NRG1-β1—which are all secreted by interstitial cells under the regulation of glucocorticoids, the hormones that primarily control surfactant maturation. To our knowledge, the present investigation is the first to document the levels of these stimulating factors over time during development of the human fetal lung. KGF displayed a higher concentration in the early-canalicular stage, coincidental with the time of ATII cell differentiation [41], than in the late-canalicular and saccular stages when these cells mature. This appears consistent with the prominent role ascribed to KGF in ATII cell differentiation [47]. Although lower, the sustained level in late pregnancy is nevertheless consistent with the stimulating activity of KGF on surfactant synthesis in fetal [9] and adult [48] type II cells. Interestingly, leptin and NRG1-β1 started increasing sharply in the human lung from about 25 wk of pregnancy, to reach their highest levels when the rate of surfactant accumulation was maximal. This is in keeping with previous animal investigations that have led the authors to consider these mediators as major paracrine regulators of surfactant synthesis [11,13]. Also in agreement is the observation of increased NRG1-β1 expression in human fetal lung explants maturing in vitro, and of enhanced surfactant synthesis by exogenous NRG1-β1 in these explants [12].

Fully consistent with findings about surfactant components, we observed no decrease for either factor in CDH lungs as compared with control lungs. KGF concentrations were even slightly higher in CDH lungs at late-gestational stages, although this difference cannot readily be explained. These data suggest that the lung hypoplasia associated with CDH does not interfere with the mesenchymal–epithelial interactions that control alveolar cell maturation. On the whole, human CDH lungs did not exhibit a trend toward a decrease in contents, or a delay in developmental changes for any of the studied parameters, including surfactant components and surfactant maturation factors.

In contrast with human lungs, KGF and NRG transcripts were decreased in the ovine model of CDH. With regard to KGF, this is in agreement with previous reports in the same model [49] and in the nitrofen model [50]. To our knowledge, NRG and leptin transcripts had not been investigated previously. Decreased expression of KGF and NRG is likely to be related to the surfactant deficit reported in the animal models. Surprisingly, TO, which is known to induce a dramatic surfactant deficit, either in the presence [16] or in the absence [51] of diaphragmatic hernia, restored KGF and NRG expression. TO also strongly up-regulated the expression of leptin. Surfactant reduction appears to be the consequence of massive TO-enhanced transdifferentiation of ATII into ATI cells [22,37,52]. TO-enhanced expression of KGF, leptin, and NRG was therefore insufficient to counteract this effect.

Although they have provided considerable insight into the understanding of the disease, animal models of CDH have shown several limitations with respect to their relevance to the human condition [53]. The present study indicates that the surfactant status of the human fetus with CDH could not be inferred from animal findings. The discordance between human CDH and the ovine model may result from the timing of the insult that occurs later in lung development in lambs and from the different degree of lung morphological maturity at full term that is more advanced in the lamb than in humans. With regard to the nitrofen model, it should be emphasized that lung hypoplasia and immaturity also occur in pups that do not develop CDH. The deficit in surfactant components, when observed, could therefore result from pulmonary toxic effects of nitrofen, including impairment of TTF1 expression, which was found to be down-regulated by nitrofen in fetal rat lungs independently of the presence of CDH [54], and also in the lung epithelial H-441 cell line [55].

Although exogenous surfactant was formerly reported to be effective in three high-risk newborns with CDH [32], a more recent multicentric study evidenced no benefit [34], consistent with our findings of normal surfactant content. Furthermore, another recent investigation concluded that late prenatal corticosteroids provide no significant benefit to fetuses with CDH [56], and this finding is consistent with a normal maturational rate and normal level of corticosteroid-dependent maturation factors as reported in this paper. By contrast, the reported efficacy of glucocorticoid therapy [57] or surfactant therapy [58] in neonatal lambs with sDH is consistent with their surfactant deficiency. However, the negative effect of TO on surfactant content [59], and the positive effects of combining maternal glucocorticoid therapy, surfactant supplementation, and TO in sheep [60], suggest possible suitability of these treatments in the care of infants treated by the PLUG method.

In conclusion, the present data challenge the paradigm that the human CDH lung is surfactant deficient as compared with the age-matched normal lung. CDH does not appear to interfere with surfactant accumulation, which occurs with normal timing and remains proportional to lung weight. Prophylactic administration of surfactant at birth in infants with CDH may therefore be useless. However, before surfactant administration can be ruled out as a potential therapy for CDH, a prospective randomized trial that also takes into account the severity of the underlying lung hypoplasia and the gestational age at delivery is necessary.

## Supporting Information

Alternative Language Abstract S1 Translation of the Abstract into French by Jacques Bourbon (30 KB DOC)

### Accession Numbers

The GenBank (http://www.ncbi.nlm.nih.gov/Genbank) accession number for the sense primer 5′-TCAGAACTTCGCATTAGCAAAGC-3′, bovine NRG–specific sequence, is NM174128. The GenBank accession number for the antisense primer 5′-GGGAGTGGACGTACTGTAGAAGCT-3′ is NM174128 for the bovine NRG–specific sequence and NM013962 for the human NRG–specific sequence.

## Abbreviations

ATII: alveolar type II
CDH: congenital diaphragmatic hernia
DSPC: disaturated phosphatidylcholine
KGF: keratinocyte growth factor
NRG: neuregulin
NRG1-β1: neuregulin 1 beta 1
PC: phosphatidylcholine
sDH: surgical diaphragmatic hernia
SP: surfactant protein
TO: tracheal occlusion
TTBS: Tris-buffered saline containing 0.05% Tween-20
TTF1: thyroid transcription factor 1
