# Supplementary material for: Surfactant Maturation Is Not Delayed in Human Fetuses with Diaphragmatic Hernia
Source: PLoS Med. 2007 Jul 31;4(7):e237. doi: 10.1371/journal.pmed.0040237 (PMC1950205; doi:10.1371/journal.pmed.0040237)
Supplement: Alternative Language Abstract S1 — (30 KB DOC) [file pmed.0040237.sd001.doc]

**Translation of the abstract into French by Jacques R. Bourbon**

**Titre**

La maturation du surfactant n’est pas retardée chez le fœtus humain avec hernie diaphragmatique

**Contexte**

La hernie de coupole diaphragmatique (HCD) est associée à une importante mortalité et morbidité chez les nouveau-nés, du fait de l’hypoplasie pulmonaire et de l’hypertension pulmonaire persistante qu’elle entraîne. L’immaturité pulmonaire globale et les études sur modèles animaux suggèrent l’existence d’une déficience en surfactant, susceptible de compliquer encore la physiopathologie de la HCD. Toutefois, les données relatives au surfactant chez le nouveau-né porteur de HCD sont contradictoires. Une limitation à la résolution de cette question réside dans l’absence d’étude chronologique de l’augmentation du contenu pulmonaire en surfactant en fin de grossesse. La pertinence d’un traitement des nouveau-nés avec HCD par du surfactant exogène reste donc une question en suspens.

**Méthodologie et Principaux Résultats**

Nous avons étudié le contenu en surfactant chez des fœtus humains porteurs de HCD par comparaison avec des fœtus de même âge gestationnel sans maladie pulmonaire. La phosphatidylcholine disaturée et les protéines du surfactant (SP) présentaient tous des concentrations similaires à un stade donné, et un même profil temporel d’accumulation au cours de la grossesse dans les deux groupes. Le thyroid transcription factor-1, un élément essentiel du contrôle de la transcription des SP, ne montrait semblablement aucune différence d’abondance entre HCD et témoins. Enfin, nous avons examiné l’expression de trois médiateurs contrôlés par les glucocorticoïdes et impliqués dans la maturation de l’épithélium alvéolaire : le keratinocyte growth factor (KGF), la leptine et la neuréguline1-β1 (NRG1-β1). Le KGF diminuait légèrement avec le temps chez les témoins, alors qu’il restait inchangé dans la HCD. La leptine et NRG1-β1 augmentaient de façon semblable en fin de grossesse dans les poumons de HCD et témoins. Ces facteurs de maturation ont aussi été déterminés chez le fœtus de mouton avec hernie diaphragmatique chirurgicale (HDc), modèle dans lequel un déficit en surfactant a été rapporté précédemment. À la différence des résultats chez l’humain, la HDc était associée à une diminution de l’expression du KGF et de la neuréguline. L’occlusion trachéale par fœtoscopie endoluminale réalisée dans ce modèle pour corriger l’hypoplasie pulmonaire a augmenté l’expression de la leptine, a partiellement restauré l’expression du KGF, et a complètement restauré celle de la neuréguline.

**Conclusions / Signification**

Nos résultats indiquent que la HCD ne perturbe pas l’accumulation de surfactant chez le fœtus humain. Les poumons de HCD ne montrent ni tendance à la diminution du contenu, ni retard dans les variations liées au développement pour aucun des composants du surfactant et des facteurs de maturation étudiés. Le contenu en surfactant est vraisemblablement approprié à la taille du poumon. Ces résultats ne sont donc pas en faveur de l’utilisation d’un traitement par le surfactant des nouveau-nés avec HCD. De plus, ils soulèvent la question de la validité des modèles animaux de HCD pour explorer la maturité biochimique du poumon.
